# Compliance with the smoking ban in urban public transportation in Chile

**DOI:** 10.18332/tid/125075

**Published:** 2020-07-22

**Authors:** Armando Peruga, Xaviera Molina, Iris Delgado, Isabel Matute, Andrea Olea, Macarena Hirmas, Claudia González, Oscar Urrejola, Ximena Aguilera

**Affiliations:** 1 Centro de Epidemiología y Políticas de Salud, Clínica Alemana, Facultad de Medicina, Universidad del Desarrollo, Santiago, Chile

**Keywords:** compliance, secondhand smoke, smoking ban, transportation

## Abstract

**INTRODUCTION:**

The aim of the study is to assess the national level of compliance with the Chilean smoke-free legislation in the urban public transportation system.

**METHODS:**

In this cross-sectional observational study, we studied a national representative sample of 475 vehicles obtained through a two-stage cluster sampling design in 2018. First, 57 municipalities were randomly selected, proportionally to the total number of public transportation vehicles. Second, within each municipality, a convenience sample of up to 4 taxis, 4 buses, and 2 metro coaches was observed. We determined the non-compliance level by systematic direct observation of smoking inside the cabin of the vehicle. We estimated the percentage of the visited vehicles where smoking was observed inside the cabin of the vehicle.

**RESULTS:**

The observation of metros, buses and taxis was completed in 24, 52, and 48, of the 57 sampled municipalities, respectively. Smoking was observed inside of about 2% of buses and 7% of taxis. Smoking was not observed in metro carriages. Overall, smoking was observed in almost 3% of the vehicles studied. A 3% noncompliance could expose a significant number of persons in public transportation to secondhand smoke, given that every 100 inhabitants results in about 84 rides a day of almost one hour duration. There are few comparable studies to put in an international context our results. In 2018, the year in which we collected the data, WHO considered that compliance with the law in public transportation was maximum. Our compliance estimate was lower, however WHO used a different methodology and its scope also included the inter-urban mobility, which we did not.

**CONCLUSIONS:**

The study highlights the need to improve the enforcement of the smoke-free law in the transportation system in Chile, which presently is almost non-existent.

## INTRODUCTION

A comprehensive smoke-free law entered into force in Chile in 2013^1^. It banned smoking in all enclosed and semi-open public areas and workplaces. It also confirmed the smoking ban in all ‘public or shared means of transportation’^2^, approved since 1995. The transportation ban affects all public means of automated mobility of passengers, including taxis^3^, in line with the World Health Organization (WHO) Framework Convention on Tobacco Control (FCTC). The guidelines of Article 8 of the WHO FCTC define public transportation as ‘any vehicle used for the carriage of members of the public, usually for reward or commercial gain. This would include taxis’^4^.

Exposure to secondhand smoke (SHS) in transportation means may be particularly harmful because they are confined spaces occupied by many people in peak hours. This is compounded by the fact that chances of encountering a smoker in Chile are significant given that 33.3% of the adults are current smokers and 20.3% report having been exposed to SHS in workplaces and public places^5^.

The purpose of this study is to assess the national level of compliance with the Chilean smoke-free legislation in the urban public transportation system. This compliance has never been measured in Chile, except for the biennial WHO assessment for their report on the global tobacco epidemic^6^, which has significant limitations and does not allow to distinguish compliance by type of vehicle.

## METHODS

### Study population and unit of observation

In this cross-sectional observational study, we studied a national sample of vehicles of the public urban passenger transportation system: taxis, buses and metro. Urban transportation moves passengers within the territory of an urban area and the immediate suburban zones. The most used means of urban transportation are buses and taxis, including collective taxis, which are taxis with fixed routes and rates. The ministry of transport reported a national fleet of 102706 taxis (58% collective taxis) and 17724 urban buses, as of December 2018^7^. Metro or light rail trains are also a common means of urban transportation in the largest urban areas.

### Sampling

A sample of 475 vehicles was obtained through a twostage cluster sampling design (Kalsbeek W. Exercise To Determine Sample Size For Precision (MOE) And Power For WHO Tobacco Law Compliance Surveys. Unpublished; 2018). First, 57 municipalities were randomly selected proportionally to the total number of existing public transportation vehicles out of the 346 municipalities existing in Chile, excluding 22 small rural ones of difficult access, representing 0.2% of the national population. Second, within each municipality, a sample of up to 4 taxis, 4 buses and 2 metro coaches were observed. This was a convenience sample, given the impossibility to obtain a complete list of vehicles with information regarding their whereabouts from where to extract a probability sample.

The national target sample size of vehicles was calculated assuming a 95% margin of error of 0.05, an expected compliance rate of 0.5, and an intra-class correlation for compliance within municipalities of 0.10.

### Outcome

The study outcome was the systematic direct observation of smoking inside the cabin of the vehicle. Smoking was defined as being in possession or control of a lit tobacco product regardless of whether the smoke was actively inhaled or exhaled.

### Data collection

#### Taxis

Fieldworkers were posted in four different intersections of transited streets of each municipality. At each intersection, the observer stood at a traffic light or stop sign, or where they could get an unobstructed observation. At each intersection, the first taxi that stopped and whose interior could clearly be seen was observed for compliance. In the few smallest municipalities, if there were no traffic lights or stop signs at the selected intersections, fieldworkers stood at a location where traffic was most likely to slow down. Also, in these municipalities, where taxis might not exist at all or in sufficient numbers to fulfil the required sample size, the observation was attempted for 30 minutes and abandoned if no taxi passed through the intersection and was not substituted.

#### Buses

The fieldworkers located a bus stop in the centre of the municipality and rode the bus line during five stops in each direction. The fieldworker repeated the operation in up to four different bus lines. In the smallest municipalities, where bus lines might be less than four, repeated observation of the same bus line was done with an interval of at least 30 minutes.

#### Metro and light rail

The fieldworkers proceeded as with bus lines, except that they rode two lines for three stops in each direction.

Observations were carried out during peak traffic hours, that is from Monday through Friday from 8 to 10 am and from 5 to 8 pm, during non-festive days, from September till December 2018.

### Data analysis

We determined non-compliance by estimating the percentage of the observed vehicles where smoking was witnessed. We present the percentage of noncompliance, by type of vehicle with their 95% confidence intervals, which are corrected for the sampling design effect. The overall smoking noncompliance was adjusted for the estimated relative weight of ridership in each studied means of transportation. Data were analysed using the STATA^©^ survey data module^8^.

## RESULTS

The observation of metros, buses and taxis was completed in 24, 52, and 48, of the 57 sampled municipalities, respectively. The lack of observed vehicles indicates that such means of transportation did not exist in the municipality at the time of the survey or their service was so sporadic that it was impossible to observe them during the window of data collection.

Table 1 shows that smoking was observed inside of about 2% of the buses and 7% of the taxis. Smoking was not observed in metro carriages. Overall, smoking was observed in almost 3% of the vehicles studied.

**Table 1:** Percentage of vehicles where a smoking violation was observed, by type of vehicle, Chile 2018

*Means of transportation*	*Municipalities without means of transportation*	*Observations*	*Smoking observed in violation of the law*	
			*%*	*95% CI*
Metro	33	65	0.0	0.0–0.0
Public bus	5	211	2.2	0.6–8.4
Taxi	9	199	6.8	2.9–15.4
Total	0	475	2.8	1.2–6.4

## DISCUSSION

Smoking violations were detected in about 3% of the urban public transport vehicles in Chile. Our study did not detect any smoking in metro carriages but did so in 2% of the buses and almost 7% of the taxis. The potential number of persons exposed to SHS in public transportation could be significant, particularly in the urban areas where 87.8% of the Chilean population resides^9^. There is no public transportation ridership data for the whole of Chile. However, a series of mobility surveys done by the Ministry of Transport, between 2010 and 2017 in the 12 most populated urban areas comprising 58% of the national population, indicated an average of 8.3 million daily person/rides in public transport or about 84 rides per 100 inhabitants^10^. The average commute time in public transportation was estimated at 54 minutes per ride in urban areas^11^. Given the ridership amount, even a small number of violations may translate into significant numbers of persons exposed to SHS.

There are not many studies of compliance with smoking bans in public transportation to put in context the results of our research. The Global Adult Tobacco Survey (GATS) conveys the proportion of the population self-reporting exposure to SHS while travelling in public transportation in the last month. Countries in the Americas that had a smoking ban in public transportation at the time of the survey reported this figure to be 4.5% in Brazil in 2008^12^, 5% in Uruguay in 2009^13^, 8% in Panama in 2013^14^, 16.3% in Mexico in 2015^15^, and 16.6% in Argentina in 2012^16^. However, Chile does not have a GATS, and these data cannot be compared with those of our study.

The WHO has evaluated compliance with the smoke-free law in Chile since 2013, including in public transportation. In 2018, the year in which we collected the data, WHO considered that compliance with the law in public transportation was maximum. Our compliance estimate was lower, even though we did not include the interurban mobility as the WHO report does. WHO’s assessment is based on the opinion of a convenience sample of three to five key observers, generally residing in the country capital and based on their experience of use of public transportation^17^. We think that ours is a more rigorous assessment based on the direct observation of a nationally representative sample of public transportation vehicles during a definite period.

### Limitations

The results of our study should be interpreted with caution. First, observing a vehicle for about 20 minutes offers a short window of opportunity to witness a smoking violation. Also, observing taxis from the outside could lead to missing some smoking violations. Therefore, noncompliance figures based on direct observation of smoking provide a conservative estimate. Second, our results are based on point prevalence estimates. They give an idea of the frequency of violations of the law but not of the duration and intensity of the exposure they produce. Therefore, it provides limited evidence of how to prioritise enforcement efforts towards vehicle types where violations may lead to more sustained exposure to SHS instead of vehicles with occasional exposure.

## CONCLUSIONS

Existing enforcement officers concentrate their efforts in inspecting mostly the hospitality sector and enforcement of the smoke-free law in urban passenger transportation seems absent^18^. Our study points to the need to direct enforcement efforts in Chile also to the transportation system.
